# The relationship between fitness and white matter using a fixel based analysis in older adults

**DOI:** 10.1093/geroni/igaf122.2755

**Published:** 2025-12-31

**Authors:** Emma Tinney, Aaron Warren, Hannah Odom, Amanda O’Brien, Edward McAuley, Arthur Kramer, Kirk Erickson, Charles Hillman

**Affiliations:** Northeastern University, Boston, Massachusetts, United States; Brigham and Women’s Hospital, Boston, Massachusetts, United States; Northeastern University, Boston, Massachusetts, United States; Northeastern University, Boston, Massachusetts, United States; University of Illinois Urbana-Champaign, Urbana, Illinois, United States; Northeastern University, Boston, Massachusetts, United States; AdventHealth Research Institute, Orlando, Florida, United States; Northeastern University, Boston, Massachusetts, United States

## Abstract

Age-related cognitive decline occurs, in part, due to diminishing white matter integrity. Higher cardiorespiratory fitness (CRF) may mitigate cognitive decline by preserving white matter structure and function, yet the mechanisms underlying these associations remain poorly understood. While prior studies have suggested that white matter microstructure mediates the relationship between CRF and cognition, they have been limited by the use of tensor-based diffusion imaging metrics, which are less sensitive to crossing fibers and complex white matter architecture. In this study, we leveraged a novel and more sensitive analytical approach, fixel-based analyses (FBA), to provide estimates of fiber density (FD), fiber cross-section (FC), and their combined measure (FDC) in multiple directions within each voxel. Using a uniquely large sample of 636 cognitively normal older adults aged 65–80 years (mean age = 69.8 years; 71% female), we hypothesized that FBA metrics would reveal robust relationships between CRF and white matter microstructure and mediate associations between CRF and cognition. In whole-brain analyses, higher CRF was associated with greater FD, FC, and FDC. Furthermore, these FBA-derived metrics statistically mediated the relationship between CRF and cognitive performance in the domains of visuospatial abilities, processing speed, working memory, and executive function/attentional control, but not episodic memory. These findings further highlight the potential for CRF to be associated with preserved cognitive function in aging through its relationship with white matter micro- and macro-structural properties, providing novel insights into the neurobiological mechanisms of fitness-related cognitive resilience.

